# Challenges in myeloperoxidase quantification: the inadequacies of current colorimetric approaches

**DOI:** 10.3389/fmolb.2025.1559499

**Published:** 2025-04-22

**Authors:** Ian Jhemes Oliveira Sousa, Kerolayne de Melo Nogueira, Ester Miranda Pereira, Rita de Cássia Meneses Oliveira

**Affiliations:** ^1^ Northeast Biotechnology Network, Focal Point on - Federal University of Piauí – UFPI, Teresina, Brazil; ^2^ Postgraduate Program in Pharmacology, Federal University of Piauí, Teresina, Brazil; ^3^ Postgraduate Program in Pharmacology, Federal University of Ceará, Fortaleza, Brazil; ^4^ Laboratory of Immunogenetics and Molecular Biology, UFPI—Federal University of Piauí, Teresina, Brazil

**Keywords:** myeloperoxidase (MPO), specificity of measurement, critical, biological marker, method

Myeloperoxidase (MPO) is an enzyme present in azurophilic compounds of neutrophils (Niknahad et al., 2016) and has been used as a quantitative marker of infiltration of these cells in inflammatory processes (Hanfer et al., 2017; Calassara et al., 2021).

MPO, in turn, catalyzes the peroxidation of chloride into hypochlorite according to the following equation:

$$H_{2}O_{2}+{\text{Cl}}^{-}+H_{3}O^{+}\;\to \text{ HOCl}+2H_{2}O$$

The generation of hypochlorite is essential for the intracellular destruction of microorganisms phagocytosed by neutrophils, which, after phagosome-lysosome fusion, hypochlorite acts as an antagonist of the protease inhibitors produced by pathogens, thus allowing lytic enzymes released by neutrophils to degrade tissues and foreign materials in the vicinity of neutrophils (Shoenfeld et al., 2007).

The measurement of MPO activity and its potential to indirectly estimate the tissue content of neutrophils in tissues has been used in scientific research associated with the investigation of inflammation mechanisms since the 1980s through colorimetric assays using chromogens such as o-dianisidine proposed by Bradley et al. (1982) or 3,3′,5,5′-tetramethylbenzidine defined by Andrews and Krinsky (1982).

In this context, the use of such tests allows the quantification of tissue MPO through the use of a homogenate, which, when incubated with the reaction solution (Tetramethylbenzidine or o-dianisidine), allows the colorimetric reaction to occur proportionally to the presence of MPO in the tissue homogenate and the values are expressed in MPO units, where a unit is usually defined the amount of MPO capable of breaking 1 mmol of peroxide/min, which allows a comparison of groups and indirect measurement of the density of polymorphonuclear cells in the tissue (Gonçalves et al., 2020).

In surveying the most recent literature from the last 5 years, it is apparent that more sensitive and specific methodologies to measure MPO activity are becoming more prevalent in the context of investigating possible anti-inflammatory activities of natural products (Mota et al., 2022; Majid et al., 2023).

We would like to point out that the last time an author expressively drew the attention of the scientific community to the nuances of MPO measurement was in 2013, when Puli et al. have searched the literature and identified 2035 studies on MPO during the previous year, of which a total of 277 measured MPO in biological matrix, for this, using the colorimetric methods described above. Puli et al., also included a brief statement on the possibility of interference from other peroxidases similar to MPO leading to skewed results (Pulli et al., 2013).

This study also importantly describes the correlation of MPO activity with the amount of tissue neutrophils and highlights the prevalence of a common error whereby authors confuse the idea of measuring MPO activity versus its presence in the tissue (Pulli et al., 2013; Mullane et al., 1985; Majid et al., 2023).

In this context, this opinion piece seeks to encourage revaluation of the established colorimetric methods for determining the presence or tissue activity of MPO and provide a brief overview of available assays with better specificity for this enzyme target. In particular, we wish to highlight the need to use the relative density of the enzyme or its enzymatic activity kinetics to represent the density of tissue neutrophils, as well as the accuracy of such measurements.

When analyzing the profile of methodologies for determining MPO activity that frequently employ substrates such as tetramethylbenzidine (TMB) and o-dianisidine, we see a great possibility of interference that can affect the specificity of the tests, since their colorimetric occurrence occurs in the face of any another type of peroxidase, which can be abundant in a complex matrix such as tissue homogenates, a situation already raised in a discussion brought by Pulli et al. (Majid et al., 2023), and corroborated by Maghzao et al. (2014).

This technical limitation is crucial because, in addition to other extracellular matrix peroxidases, several defense cells have similar peroxidases, such as eosinophil peroxidase (EPO), which, according to the high-resolution (1.6 Å) structure characterization by Pfanzagl et al. (2023), “made it possible to identify differences that may contribute to the known divergent enzymatic properties”, but when observing this statement, we cannot fail to address the fact that they are still peroxidases and therefore catalyze similar chemical reactions, which, according to White et al. (1991) MPO and EPO may interfere with each other in the measurement of their activities by colorimetric methods.

Furthermore, it is important to emphasize that MPO is not exclusive to neutrophils, but is also expressed in other granulocytes, such as monocytes (Bos et al., 1978; Gurski and Dittel, 2022). The prevalence of this enzyme in other cell types suggests that MPO may be involved in granulocyte cellular processes other than neutrophil migration.

Thus, the diversity of cells expressing MPO and other similar peroxidases creates a level of complexity which further reduces the precision and accuracy of assays founded on colorimetric methods based on generic chemical reactions. The potential for drawing erroneous conclusions in dangerously high, especially when using said assays to characterize pathological conditions such as cardiovascular, neurodegenerative and autoimmune diseases.

Given the preclinical relevance of MPO measurement for prospecting biological properties (Huang et al., 2016), it is essential that the quantitative methodologies used for its determination and/or its activity be reviewed and improved, and that tests using traditional colorimetric methodologies mention that the activity evaluated is of total peroxidases or even perform a double assay with subtraction of the background activity using the specific myeloperoxidase inhibitor (Rawlins et al., 2007).

A useful and inexpensive solution would be to adopt more advanced detection techniques, such as enzyme-linked immunosorbent assays (ELISA) using antibodies specific for MPO or neutrophil differentiation clusters, which could provide a strong and linear response to the presence of polymorphonuclear cells (Spijkerman et al., 2020). These methods, in addition to providing greater specificity in the detection of MPO, would also facilitate more accurate quantification of the tissue content of neutrophils in lien with their true biological expression.

On the other hand, when using methodologies that measure the presence of the MPO enzyme, authors should be aware that their result has a strong positive correlation with the presence of neutrophils in the tissue, but has no connection with the activity of the enzyme itself.

Considering the importance of identifying neutrophil activity and not just their presence in tissues, a more refined approach would involve the use of markers that are internalized specifically by MPO-expressing cells and that only yield a signal after activation of the enzyme. The basis for the development of such assays is already in place with receptors such as CD11b, CD66b, and CD64 already classified as markers of neutrophil activation (Prausmüller et al., 2022).

It is our hope that in highlighting these shortcomings in established methods for MPO activity quantification we can prompt reconsideration of primitive colorimetric techniques and inspire development of novel highly-specific protocols. In our view, it is essential that the scientific community recognizes and addresses the need to improve methodologies for determining MPO activity and/or its tissue content to avoid misleading scientific insights that may influence the direction of preclinical and clinical scientific research.
